# A facile strategy for preparation of Fe_3_O_4_ magnetic nanoparticles using *Cordia myxa* leaf extract and investigating its adsorption activity in dye removal

**DOI:** 10.1038/s41598-023-50550-1

**Published:** 2024-01-02

**Authors:** Elham Ghoohestani, Fayezeh Samari, Ahmad Homaei, Saeed Yosuefinejad

**Affiliations:** 1 Department of Chemistry, Faculty of Sciences, University of Hormozgan, P.O. Box 3995, Bandar Abbas, Iran; 2 Nanoscience, Nanotechnology and Advanced Materials Research Center, University of Hormozgan, Bandar Abbas, Iran; 3 Department of Marine Biology, Faculty of Marine Science and Technology, University of Hormozgan, Bandar Abbas, Iran; 4 Research Center for Health Sciences, Institute of Health, Department of Occupational Health Engineering, School of Health, Shiraz University of Medical Sciences, Shiraz, Iran

**Keywords:** Chemistry, Nanoscience and technology

## Abstract

This study demonstrates the successful, facile, and cost-effective preparation of magnetic Fe_3_O_4_ nanoparticles (MNPs) via green procedure using *Cordia myxa* leaf extracts for efficient adsorption of methylene blue (MB) as a model of organic pollutant. The formation of Fe_3_O_4_ NPs was confirmed by a range of spectroscopy and microscopy techniques including FT-IR, XRD, FE-SEM, TEM, EDS, VSM, TGA, and BET-BJH. The synthesized spherical nanoparticles had a high specific surface area of 115.07 m^2^/g with a mesoporous structure. The formed Fe_3_O_4_ MNPs exhibited superparamagnetic behavior with saturation magnetization of 49.48 emu/g. After characterization, the adsorptive performance of the synthesized MNPs toward MB was evaluated. To achieve the maximum removal efficiency, the effect of key parameters such as adsorbent dosage (MNPs), initial adsorbate concentration, pH, and contact time on the adsorption process was evaluated. A maximum adsorption capacity of 17.79 mg/g was obtained, after one-hour incubation at pH 7.5. From the pH_PZC_ of 7.1 of the synthesized adsorbent, the electrostatic attraction between MB and Fe_3_O_4_ NPs plays an important role in the adsorption process. The adsorption experimental data showed the closest match with the pseudo-second-order kinetic and Langmuir isotherm. The prepared Fe_3_O_4_ NPs were easily recovered by an external magnet and could be reused several times. Therefore, the synthesized MNPs seem to be excellent adsorbents for the removal of MB from aqueous solution.

## Introduction

The release and permeation of toxic organic compounds and synthetic dyes to surface water can cause serious problems and harm to the environment and living things. Dye effluents are released from numerous dye-utilizing industries such as paper, textile, painting, cosmetics, plastics, printing, rubber, and pharmaceutical manufacturing^1–6^. The complex chemical structure of synthetic dyes especially the existence of aromatic rings in their molecular structure causes high durability without any volatility in high temperature, high solubility, stability, and non-biodegradability^7,8^. These dyes are very poisonous and may result in genetic mutation, cancer, allergic reactions, skin problems, respiratory tract diseases, heart problems, and toxicity effects of neurotoxicity, which pose serious harm to human health^5^. Thus, it is recommended that dye wastewater should be treated and organic dyes be correctly disposed of before being released into the aquatic environment to minimize its negative impacts and maintain ecosystems^6,9^. Due to the importance of the removal of dye from wastewater, finding more efficient, cheaper, and safer ways to remove dyes from industrial wastewater has become a focal point for environmental researchers^10,11^. Till now, several approaches have been applied for removing these hazardous dyes from the water/wastewater such as adsorption, precipitation, chemical oxidation, biological removal, membrane filtration, and coagulation–flocculation^4,9^. Among them, the adsorption technique is the most preferable treatment method for a wide range of water contaminants due to its low operational cost, simplicity of design, efficiency, and higher selectivity, regeneration, and designability^5^. In addition to these advantages of the absorption method, the use of simple, economical, and environmentally friendly adsorbents is one of the important issues in these technologies. Currently, several inexpensive adsorbents are available, such as clays^12^, zeolites^13^, plant waste materials^14^, and magnetic nanoparticles^15^.

Metal oxide nanoparticles have recently attracted much attention because of their interesting physical, chemical, and catalytic characteristics ^16–18^. Among them, Fe_3_O_4_ nanoparticles (MNPs) have received special attention due to their magnetic and microwave absorbing properties, their low toxicity, high stability, low cost, and simple production^10,19^ These properties have made Fe_3_O_4_ MNPs suitable for application in various areas, such as in the chemical, physical, biological, medicinal, and material sciences. For instance, MNPs are utilized for data storage, drug delivery systems, catalysts, magnetic resonance imaging, and tissue repair. Also, the MNPs have attracted considerable interest in water treatment due to their high magnetic response-ability, nanoscale particle size consequently large surface area, superb adsorption, high magnetization, surface functionality, and biocompatibility^20,21^. Considering the importance of MNPs, a wide variety of chemical and physical procedures have been applied for the preparation of Fe_3_O_4_ MNPs, such as solvothermal synthesis^22^, hydrolysis^23^, sonochemical synthesis^24,25^, hydrothermal synthesis^26^, electrochemical synthesis ^27^, inverse emulsion polymerization^28^, surfactant-templated synthesis^29^, sol–gel technology^30^, laser ablation^31^, and co-precipitation^32^. However, such methods faced limitations of toxic chemicals/reagents, sophisticated procedures, temperature requirements, and energy consumption, and therefore they affect the environment^33,34^. To circumvent the problems of conventional synthesis procedures, biosynthesis of nanoparticles has recently been introduced as an efficient alternative to physicochemical methods since it is affordable, simple, and environmentally friendly, without requiring hazardous chemicals and producing toxic by-products ^35,36^. The biosynthesis of Fe_3_O_4_ MNPs can be performed by numerous organisms such as yeasts^37^, algae^38^, bacteria^39^, fungi^40^, and plants^8,20,41^. Meanwhile, plant extracts are usually easy to handle, and the process can easily be scaled up, with good control of the synthesis and stabilization of MNPs^33^. Plant extracts contain phytochemicals such as terpenoids, flavonoids, alkaloids, and phenolic compounds, which act as reducing agents as well as stabilizing the MNPs^42,43^. Depending on which plant extracts are used, the produced nanoparticles may differ in size, morphology, dispersity, and capping materials, and hence may differ in their physical, chemical, and biological activities^44^. Therefore, it is of interest to study the effects of different plant extracts on the characteristics of the obtained nanoparticles.

From above, the main goal of this work was the synthesis of Fe_3_O_4_ MNPs with an easy and green approach using aqueous leaf extract of *Cordia myxa* through a simple co-precipitation route without the need for hazardous chemicals and investigation of its adsorption efficiency in the removal of MB. MB is a cationic dye classified as a toxic dye and could result in some health and environmental problems^8^. *C. myxa*, commonly known as Assyrian plum or Sebesten, is native to an area stretching from tropical Africa through the Middle East^18,45^. It possesses painkilling, laxative, anti-inflammatory, antimicrobial, immunomodulatory, antiparasitic, and insecticidal properties^46,47^. It contains various phytochemicals such as glycosides, flavonoids, tannins, sterols, saponins, phenolic acids, terpenoids, and alkaloids^45^. In this introduced synthesis of Fe_3_O_4_ MNPs, aqueous leaf extract of *C. myxa*, containing phenolics and antioxidants, was used as a reducing and stabilizing agent. After characterization by various biophysical techniques, the synthesized Fe_3_O_4_ MNPs were applied as a sorbent, due to the easy collection and effective separation of adsorbents using an external magnetic field, for removing methylene blue (MB) as an organic model dye in aqueous media. Moreover, the adsorption and kinetics isotherms were identified. The prepared MNPs showed excellent MB adsorption capacity, and they could easily be recycled from the target reactor by utilizing an external magnetic field.

## Materials and methods

### Chemicals and materials

Ferric chloride hexahydrate (FeCl_3_.6H_2_O) and ferrous sulfate heptahydrate (FeSO_4_.7H_2_O), methylene blue (MB), all with analytical grades, and sodium hydroxide (NaOH, > 97.0%) were purchased from Merck chemical company (Germany). All chemicals used in synthesis or application steps were of analytical reagent grade and utilized as received. In the synthesis procedures, the distilled was of deionized grade. All glassware was thoroughly cleaned with aqua regia and rinsed with deionized water.

### Collection of the plant and preparation of the extract

This study complies with relevant institutional, national, and international guidelines and legislation. Fresh and healthy *Cordia myxa* L. leaves were collected from Bandar Abbas, Hormozgan province, Iran (57° 33′ E 227° 30′ N, 1050 m). The guidelines for collecting plants botany 440/540 (available at https://herbarium.eku.edu/) were used for plant collection. Dr. Mansoore Shamili (Horticulture Department, University of Hormozan, email: shamili@ut.ac.ir) identified the plant species. Accession number 386 was assigned to the plant sample in the university herbarium. Fresh *Cordia myxa* L. leaf extract was prepared via the green procedure which means not using any toxic or dangerous chemical additives. No cultivation of the plant was done, and there was no genetically modified organism (GMO) procedure. The collected leaves were exhaustively washed with tap water and deionized water to remove surface pollutants and dust particles. Drying the leaves was done in the shade at room temperature for 10 days. A powder was prepared from the dried leaves via an electric blender. 10.0 g of the powdered leaf was added to a 250 ml round-bottom flask containing 100 ml deionized water and refluxing was done for 60 min at 80 °C. After cooling, the mixture was filtered using Whatman No. 1 filter paper, and the final extract was kept in a refrigerator for the next characterization or studies.

### Synthesis of Fe_3_O_4_ MNPs

The preparation of Fe_3_O_4_ MNPs was conducted via the following easy and environmentally-friendly procedure. In a typical reaction, 1.11 g of FeCl_3_.6H_2_O (0.004 mol) and 0.53 g of FeSO_4_.7H_2_O (0.002 mol) (amounting to a Fe^3+^: Fe^2+^ molar ratio of 2:1) were added into 100 ml deionized water, and heated to 80 ℃ under atmospheric pressure under mild stirring using a magnetic stirrer for 10 min. 5.0 ml of freshly prepared *C. myxa* leaves extract was added, and, under vigorous stirring for 1 h at 80 °C, 20.0 ml 1 M NaOH solution was added dropwise. During this step, the pH of the reaction was kept at pH 10–11. The color of the prepared mixture changed from yellow to completely black, which was the indicator of the construction of Fe_3_O_4_ MNPs in the precipitation reaction^41^. After cooling, the isolation of synthesized Fe_3_O_4_ MNPs was performed via an external magnet, and the MNPs were washed several times with ethyl alcohol. The separated Fe_3_O_4_ MNPs were dried in a vacuum oven at 90 °C for 12 h. For comparison, Fe_3_O_4_ MNPs were also synthesized in the absence of plant extract using a similar procedure. The obtained MNPs were stored in a stoppered bottle until further use.

### Instrumentation and characterization

The obtained Fe_3_O_4_ MNPs were analyzed by X-ray powder diffraction (Bruker D8 Advance powder diffractometer) with Ni-filtered Cu-*K*_*α*_ radiation (λ = 1.5406 Å) at a setting of 40 kV/30mA with a scan rate of 0.02° per minute in the angular range (2θ) of 20 to 70°. FTIR spectra of *C. myxa* leaves extract and synthesized Fe_3_O_4_ MNPs were obtained in the range of 4000–550 cm^−1^ using a Bruker alpha FT-IR spectrometer (Germany) equipped with a Diamond attenuated total reflection (ATR) accessory at room temperature. The morphologies of the samples were observed using field emission scanning electron microscope images (FE-SEM) on a TE-SCAN MIRA3 SEM with primary electron energy of 15 kV. The chemical composition of the obtained Fe_3_O_4_ MNPs was investigated by Energy Dispersive X-ray Spectroscopy (EDS) performed in SEM. TEM studies were carried out on a Zeiss–EM10C instrument with an accelerating voltage of 100 kV. The magnetic properties of the synthesized Fe_3_O_4_ MNPs were identified at room temperature using a vibrating sample magnetometer (VSM; Meghnatis Daghigh Kavir Company LBKFB). The pore diameter and specific surface area were measured using a Brunauer–Emmett–Teller surface area analyzer (Microtracbel Corp BELSORP Mini). Nitrogen adsorption measurements were done on samples that were priory degassed at 150°C. Thermogravimetric analysis (TGA) was done with a heating program from room temperature up to 700 ºC with an increasing slope equal to 10 °C min^−1^ under a nitrogen atmosphere using a TGA-7 analyzer (Perkin-Elmer, USA). UV–Vis spectra were obtained at room temperature with a SCINCO-S-3100 spectrophotometer (Scinco Co., Korea) equipped with a 1.0 cm quartz cell.

### Point of zero charge (PZC)

Determination of the point of zero charge (pH_PZC_) for the synthesized Fe_3_O_4_ MNPs was done using the pH drift method^48^. It is well-known that the pH_PZC_ is the pH at which the surface charge of the synthesized Fe_3_O_4_ MNPs is equal to zero. To a series of 100 ml falcon tubes, 50.0 ml of 0.01 M NaCl (as an inert electrolyte) was added, for adjusting the ionic strength throughout the experiments. Next, the pH values of the solutions (pH_initial_) were brought to a pH value in the range between 2.0 and 10.0 with intervals of one by adding either HCl or NaOH. 0.010 g of the synthesized Fe_3_O_4_ MNPs was then added to each falcon tube and they were closed. The resulting mixtures were allowed to equilibrate for 12 h in a shaker kept at room temperature. After 12 h, the synthesized Fe_3_O_4_ MNPs were magnetically separated from the solution and the pHs of the remaining solutions were measured (pH_final_). The plot of ∆pH (pH_final_−pH_initial_) versus pH_initial_ was drawn and the point of pH_final_−pH_initial_ = 0 was considered as the pH_PZC_ value^49^.

### Batch experiments

To evaluate the adsorption of methylene blue by the synthesized Fe_3_O_4_ MNPs, 15 mg of Fe_3_O_4_ MNPs were stirred for 2 h in 30.0 ml of MB solution (10 mg L^−1^ in distilled water) in a shaker at room temperature. The reaction was monitored by UV–Vis spectrophotometer at time intervals of 10 min by taking 3.0 ml of the mixture and separating the Fe_3_O_4_ MNPs from the reaction solution through an external magnet. The MB concentration remaining in the reaction mixture was determined by UV–Vis spectrophotometry at a wavelength of 664 nm. The precision of UV–Vis measurements before and during work was checked with a spectrophotometer, and the RSD of absorbance was not higher than 3%. The efficiency of the Fe_3_O_4_ MNPs in MB removal was obtained using Eq. (1)^50^:

1 $$R=(\frac{{A}_{0}-{A}_{t}}{{A}_{0}})\times 100$$

where *A*_0_ is the initial absorbance of MB in the solution and *A*_*t*_ is its absorbance at time *t*.

Effects of several experimental factors on the adsorption efficiency of MB by Fe_3_O_4_ MNPs including pH (3.0, 5.0, 6.5, 7.5, 9.0, and 11.0), amount of Fe_3_O_4_ MNPs (0.17, 0.25, 0.33, 0.50, 0.67, and 0.83 mg ml^−1^), and initial MB concentration (5.0, 10.0, 12.0, and 15.0 mg L^−1^) were investigated. Each experiment was performed three times and the mean ± SD was reported. The adjustment of the pH was performed using HCl or NaOH solutions as required.

The capacity of the synthesized Fe_3_O_4_ MNPs to adsorb MB was calculated using Eq. (2)^41,51^:

2 $${q}_{t}=({C}_{0}-{C}_{t})\frac{V}{m}$$

where *q*_*t*_ is the adsorption capacity per gram of magnetic adsorbent (synthesized Fe_3_O_4_ MNPs) at desired time *t*. *C*_0_ and *C*_*t*_ denote the dye concentrations (mg L^−1^) in the aqueous phase at the start of the experiment and at the desired time (*t)*, respectively. The volume of the dye solution (L) is also shown by *V* in Eq. (2) and *m* denotes the mass (g) of the magnetic adsorbent. It is worth mentioning that at *t* in equilibrium contact time, *C*_*t*_ is equal to *C*_*e*_ and *q*_*t*_ is equal to *q*_*e*_ respectively.

## Results and discussion

### Powder XRD analysis

**Figure 1:**
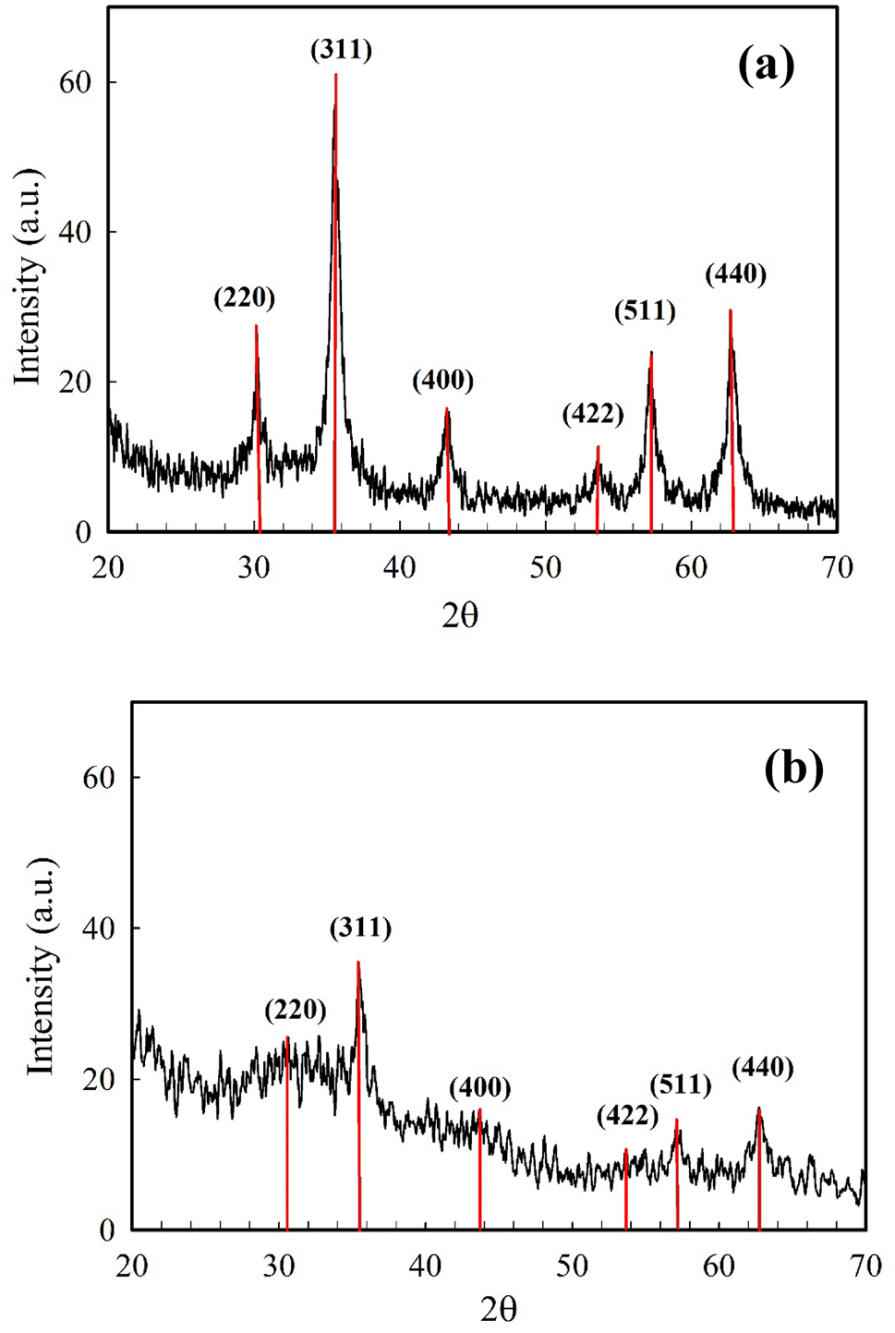
X-ray powder diffraction pattern of (**a**) Fe_3_O_4_ MNPs synthesized in the presence of *C. myxa* extracts and (**b**) without using the *C. myxa* extracts.

The crystallinity of the synthesized Fe_3_O_4_ MNPs was characterized by X-ray powder diffraction (XRD) (Fig. 1a). XRD analysis showed six major Bragg diffraction peaks at 30.24°, 35.64°, 43.42°, 53.52°, 57.26° and 63.68° (2θ), which correspond to the (220), (311), (400), (422), (511) and (440) crystal indices (corresponds to the standard XRD pattern of Fe_3_O_4_ from JCPDS 75-0033)^52^. These diffraction peaks are very similar to the peaks of the cubic spinel structure of Fe_3_O_4_ crystals^52,53^. No other distinct peaks of metal hydroxides or α-Fe_2_O_3_ (hematite) were observed, indicating the pure crystalline phase of the synthesized Fe_3_O_4_ MNPs and confirming the complete formation of Fe_3_O_4_ MNPs. The average crystallite size of the as-prepared Fe_3_O_4_ MNPs was estimated from the full width at half-maximum (FWHM) of the (311) reflection peak using Debye–Scherrer’s equation (Eq. 3)^35^:

3 $$D=\frac{0.89\lambda }{\beta cos\theta }$$

In Debye–Scherrer’s equation, 0.89 is the shape factor, *D* denotes the average particle size and *λ* is the wavelength of the Cu-*Kα* irradiation. *β* shows the full width at half maximum intensity of the obtained diffraction peak and *θ* is the diffraction angle for the (311) peak of the Fe_3_O_4_ MNPs. The calculated crystallite size of the Fe_3_O_4_ MNPs was ~ 25.3 nm. To ensure the ability of the chemical method used to prepare MNPs without using the plant extract, the XRD of Fe_3_O_4_ MNPs prepared in the absence of *C. myxa* leaf extract is also represented in Fig. 1b. According to the results, the indicative peaks of MNPs in the XRD pattern show the construction of desired particles.

### FE-SEM, EDS, and TEM of Fe_3_O_4_ MNPs

**Figure 2:**
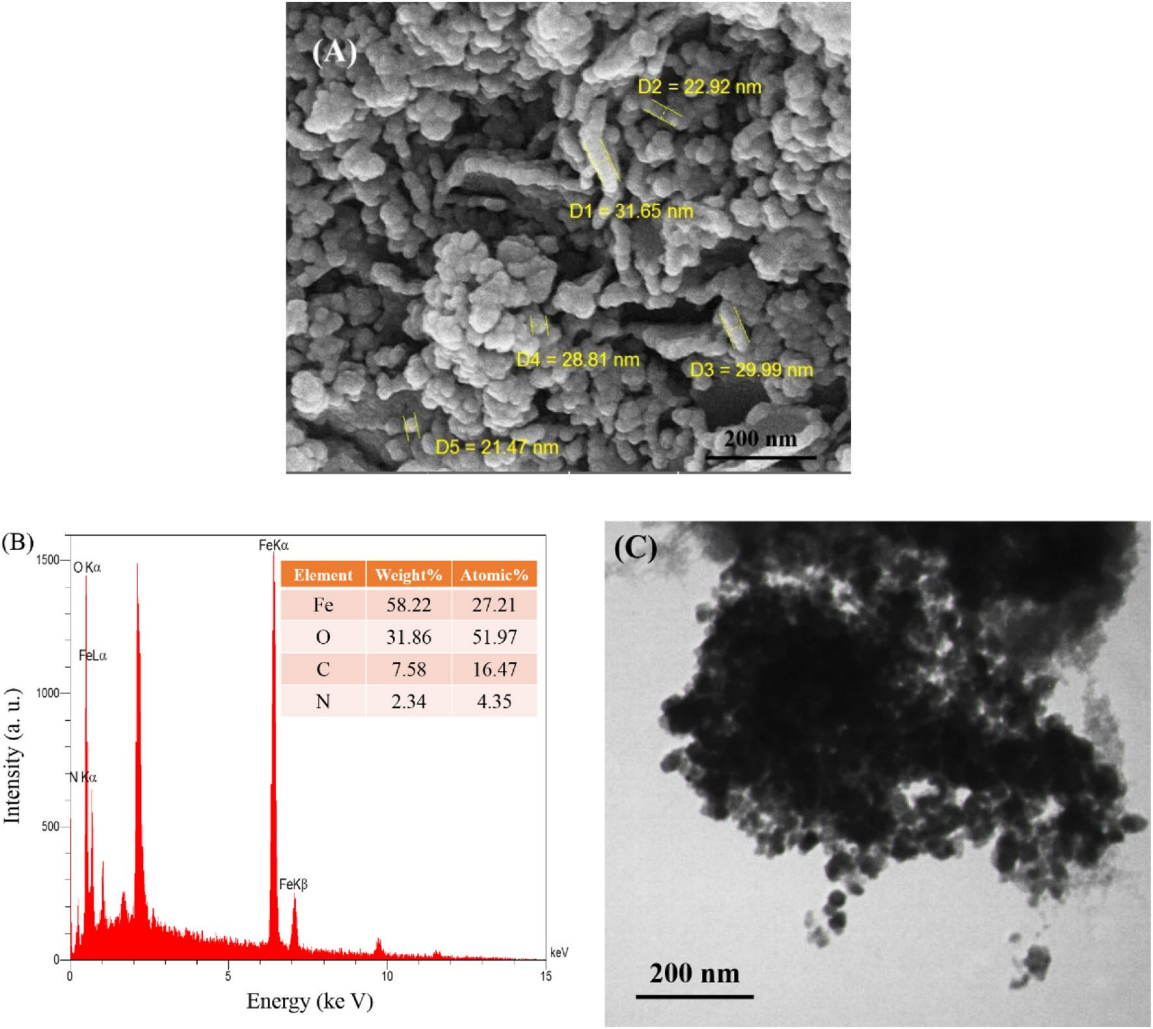
(**A**) Field emission scanning electron microscope image, (**B**) Energy Dispersive X-ray Spectroscopy spectrum, and (**C**) Transmission Electron Microscopy image of the synthesized Fe_3_O_4_ MNPs.

The size and shape of synthesized Fe_3_O_4_ MNPs were established by FE-SEM. As can be seen in Fig. 2a, the synthesized nanoparticles were spherical in shape and uniform in size with a size range of 21–32 nm, which was similar to the XRD result.

The composition of the Fe_3_O_4_ MNPs was established by EDS in SEM, showed that iron, oxygen, carbon, and nitrogen are the four main elements with weight percentages of 58.22, 31.86, 7.58 and 2.34%, respectively (Fig. 2b). The elements of carbon and nitrogen were derived from the phytochemicals found in *C. myxa* leaf extract. The presence of these elements proves that the prepared nanoparticles are coated with phytochemicals^54^. Also, the source of excess oxygen can be from flavonoids and phenolics of *C. myxa* leaf extract or the physical adsorption of oxygen from air on the surface of synthesized Fe_3_O_4_ MNPs. Furthermore, TEM images of the prepared nanoparticles confirmed the formation of spherical particles (Fig. 2c).

### FTIR analysis

**Figure 3:**
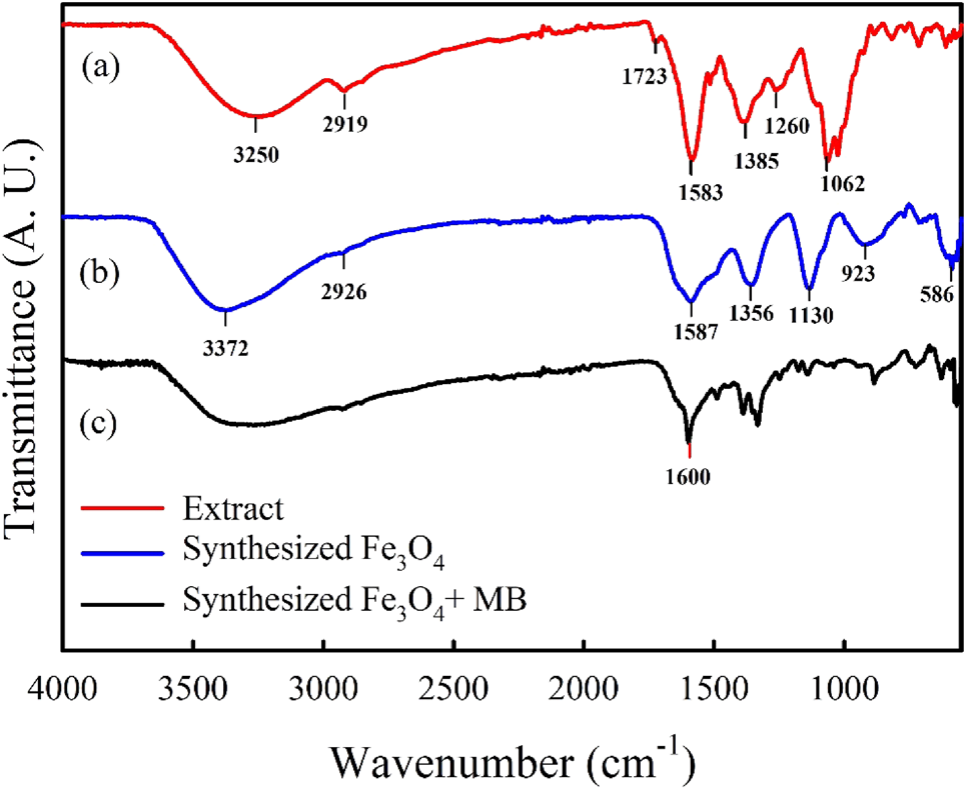
FT-IR spectrum of (**a**) *C. myxa* leaf extract, (**b**) synthesized Fe_3_O_4_ MNPs, and (**c**) synthesized Fe_3_O_4_ MNPs after adsorption of MB.

**Figure 4:**
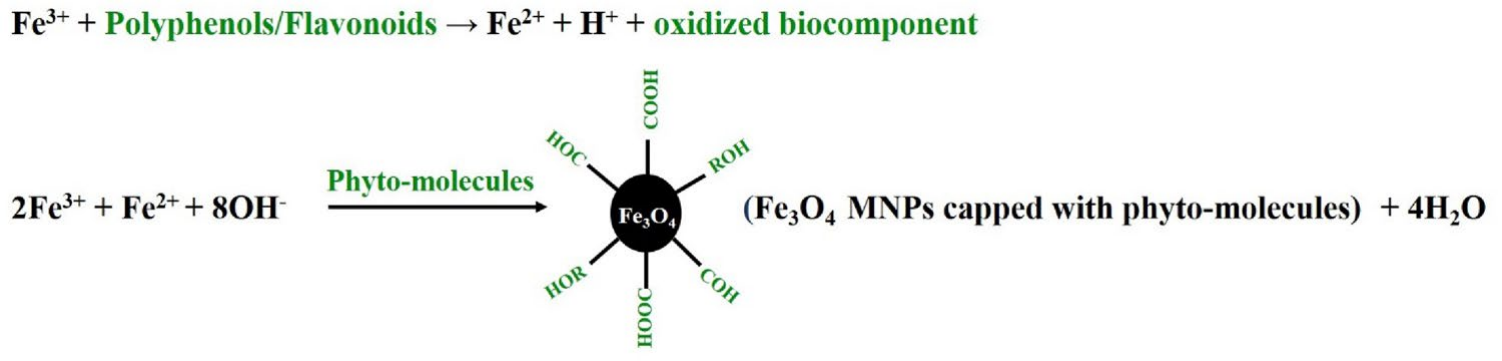
Proposed mechanism for synthesis of Fe_3_O_4_ MNPs in the presence of C. myxa extract.

FT-IR analysis was performed to identify possible functional groups of the *C. myxa* leaf extract on the surface of prepared magnetic nanoparticles. The FT-IR spectrum of *C. myxa* leaf extract (Fig. 3a) displays peaks at 3500 to 3000 (centered at 3250), 2919, 1723, 1583, 1385, 1260, and 1062 cm^−1^, corresponding to free OHs and OH group forming hydrogen bonds, aliphatic C–H stretching vibrations, aromatic ring C=C stretching vibrations, amide C=O stretching vibrations, nitrogen N–O bending vibrations, C–OH stretching vibrations, and C–N stretching vibrations of amine groups, respectively^3,4,55,56^. These peaks indicate the presence of flavonoids and phenolics in *C. myxa* leaf extract, which could reduce metal ions to metal nanoparticles and stabilize the formed nanoparticles^57^. In Fig. 3b, the peak at 586 cm^−1^ is the characteristic Fe–O peak, confirming the successful formation of Fe_3_O_4_ MNPs^3,4,10,56^. Furthermore, the differences between the FT-IR spectrum of the *C. myxa* leaves extract and the synthesized Fe_3_O_4_ MNPs indicate that the iron cations interact with the phytochemicals (Fig. 3b). The shifted peaks at about 3372, 2926, 1587, 1356, 1130, and 923 cm^−1^ correspond to the O–H functional groups, C–H stretching, C=O stretching, nitrogen N–O bending, and C–N stretching vibrations, respectively^50^. FT-IR results indicate that the flavonoids and phenolics in *C. myxa* leaf extract act as capping agents for the formed Fe_3_O_4_ MNPs and prevent their aggregation through surface adsorption via π-electron interaction in the absence of other strong capping agents. A possible mechanism for the formation of Fe_3_O_4_ MNPs can be proposed as follows^58^ and is also illustrated in Fig. 4 to highlight the role of functional groups that modify the surface of MNPs:

The FT-IR spectrum of the modified Fe_3_O_4_ MNPs after adsorption of MB is also shown in Fig. 3c. By comparing this spectrum with the spectrum of modified Fe_3_O_4_ MNPs (Fig. 3b), it can be observed that the C=C bond (located around 1600 cm^−1^) in Fig. 3c is slightly reduced compared to Fig. 3b, but this change was not significant after MB adsorption. Due to the repetition of this fact in different runs, it can be suggested that the contribution of π–π interaction is not significant in this interaction^59^. On the other hand, the reduction of C–O–H bond at about 1070 cm^−1^ has a significant loss in intensity after MB adsorption which indicates the possibility of electrostatic interactions and H-bonding during this phenomenon.

### Magnetic measurements

**Figure 5:**
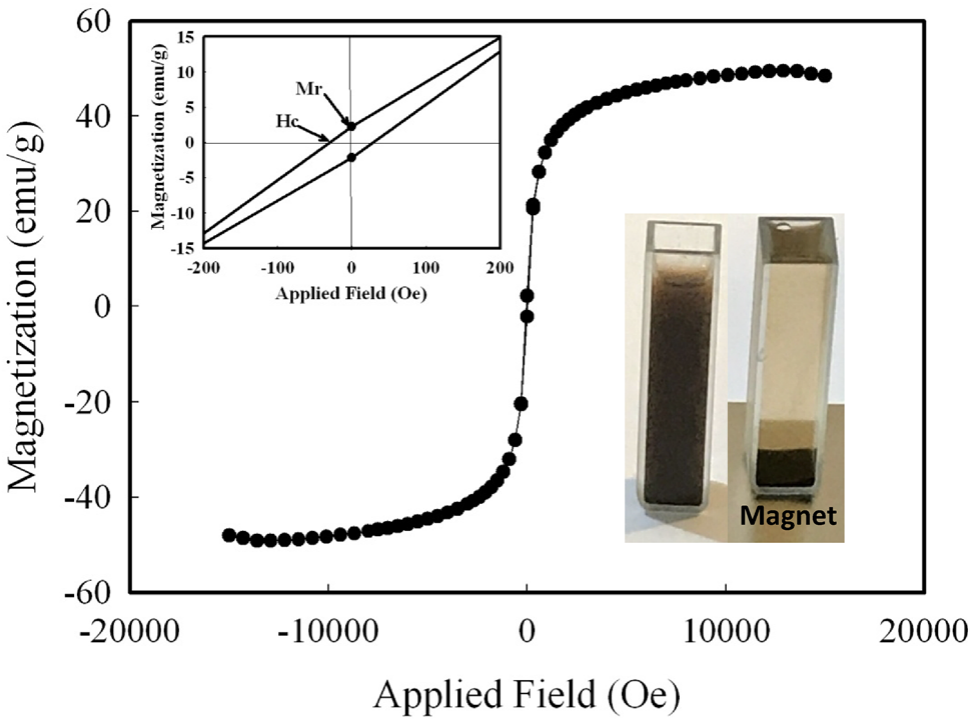
Room temperature magnetization–hysteresis (M–H) loops of synthesized Fe_3_O_4_ MNPs. The inset (upper-left) is an enlarged hysteresis loop; the lower-right inset shows M–H loops of the solution before and after separation by an external magnet.

The shape, size, and morphology of nanomaterials, which are strongly dependent on the applied synthetic method, could affect the magnetic behavior of the nanomaterials^55^. Therefore, the magnetic properties of the prepared Fe_3_O_4_ MNPs were investigated at room temperature via a vibrating sample magnetometer (VSM), with a field sweeping from − 15,000 to + 15,000 Oe. Figure 5 shows the superparamagnetic behavior of the synthesized Fe_3_O_4_ as the magnetic hysteresis loop shows an S-like curve^60^. The observed saturation magnetization (Ms) was ~ 49.48 emu/g. The remnant magnetization (Mr) and coercivity (Hc) of the synthesized Fe_3_O_4_ MNPs were 2.25 emu/g and ~ 30 Oe, respectively (upper left inset of Fig. 5). These low values of Mr and Hc indicate the superparamagnetic behavior of the synthesized MNPs^53^. Moreover, the sufficient saturation magnetization of the synthesized Fe_3_O_4_ MNPs allowed easy and rapid separation (within seconds) of these MNPs from the mixture by an externally applied magnet, with the solution becoming clear (bottom right inset of Fig. 5). This property is very important in the reusability of the sorbent.

### Thermogravimetric analysis

To investigate the thermal stability of the prepared Fe_3_O_4_ MNPs, a thermogravimetric analysis (TGA) of Fe_3_O_4_ MNPs with and without leaf extract was done (Fig. S1a,b), supporting information). The synthesized Fe_3_O_4_ MNPs in the presence of leaf extract show three weight loss steps (Fig. S1a). In the first step (below 100 °C), the weight loss is due to the dehydration of the sample, the remaining weight of which is about 96%. The weight loss steps at 230 °C and at 420 °C can be attributed to the decomposition of adsorbed phyto-compounds of *C. myxa* leaf extract that act as capping agents^55^. At temperatures higher than 520 ºC, the phytochemicals of *C. myxa* leaf extract were completely degraded, and the MNPs did not show further weight loss up to 700 ºC. For the MNPs caped with C*. myxa* leaf extract, the residual weight is 86% after 520 ºC (Fig. S1a). In contrast, the residual weight of the Fe_3_O_4_ MNPs synthesized without plant extract is about 96% at 520 ºC to 700 ºC (Fig. S1b), which is close to the residual weight of the Fe_3_O_4_ MNPs synthesized with leaf extract after the first step of weight loss. These results indicate that the Fe_3_O_4_ MNPs synthesized without extract contain only adsorbed water without any capping agent.

### Surface area and pore distribution

The surface area and porous nature of the synthesized MNPs were investigated by determining the adsorption–desorption isotherm at 77 K using liquid N_2_ as adsorbent, as shown in Fig. S2 (Supporting information). The synthesized Fe_3_O_4_ MNPs exhibited hysteresis loops with intensities associated with capillary condensation at relatively high pressures, which are characteristic of type IV isotherms with H3 type hysteresis loops, according to the IUPAC classification^10,61^. The calculated Brunauer–Emmett–Teller (BET) surface area of the prepared green-coated MNPs was about ~ 115.07 m^2^/g, which is clearly higher than that of many other Fe_3_O_4_ MNPs^10^. The single-point adsorption total volume at P/P_0_ = 0.990 was 0.3357 cm^3^ g^−1^. The values of surface area and pore volume of the synthesized Fe_3_O_4_ MNPs indicate the potential of the proposed method in preparing the Fe_3_O_4_ MNPs with superior catalytic or adsorption activity. Moreover, the pore size distribution from the Barrett-Joyner-Halenda (BJH) analysis (inset of Fig. S2), indicates the mesoporous nature of the synthesized Fe_3_O_4_ MNPs, with a wide pore size distribution. Overall, the high BET-specific surface area and the BJH pore-size distribution analysis confirmed that these one-pot synthesized mesoporous Fe_3_O_4_ MNPs have the potential to be used for the adsorption of pollutants such as dyes and toxic metals from wastewater.

### Adsorption of methylene blue

Next, the synthesized Fe_3_O_4_ MNPs were used to remove MB, as a model of an organic dye pollutant, from aqueous solution. The effects of various parameters on the adsorption capacity of the Fe_3_O_4_ MNPs were followed: including the pH of the solution, the amount of sorbent, the dye concentration, and the adsorption time.

#### pH dependence studies

**Figure 6:**
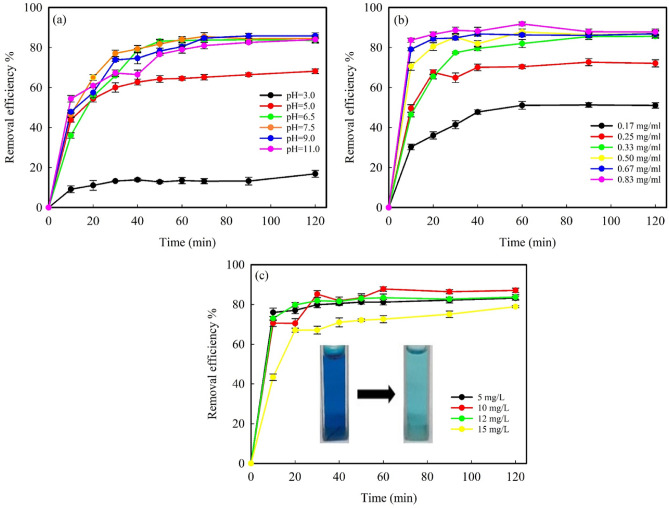
Adsorption efficiency of the synthesized Fe_3_O_4_ MNPs versus time as a function of (**a**) solution pH (other experimental conditions: MNPs dosage = 0.33 mg ml^−1^ and MB conc. = 10.0 mg L^−1^), (**b**) concentration of synthesized Fe_3_O_4_ MNPs (at pH = 7.5 and MB conc. = 10.0 mg L^−1^), and (**c**) initial MB concentration (at pH = 7.5 and MNPs dosage = 0.50 mg ml^−1^). Inset: The color change of MB solution with the concentration of 12.0 mg L^−1^ before and after the adsorption process (at the optimum pH of 7.5 and adsorbent dosage of 0.50 mg ml^−1^).

The pH of the solution is a key factor in the adsorption of dye from water because pH affects the surface charge of the adsorbent as well as the structure and ionization value of the dye molecules^62^. Using an initial dye concentration of 10 mg L^−1^ and 10 mg MNPs in 30.0 ml dye solution (0.33 mg ml^−1^) the removal efficiency of MB by Fe_3_O_4_ MNPs was studied at pHs from 3.0 to 11.0. According to Fig. 6a, the removal efficiency increases with the increase of initial pH solution (from pH 3.0 to 7.5) and remains almost constant at higher pHs. Similar remarks have been observed in the adsorption of methylene blue by other adsorbents^63–65^. This result can be explained by the pH_PZC_ value of the adsorbent. In pH_PZC_, the electric charge density on the sorbent surface immersed in the electrolyte solution is zero. At pH < pH_PZC_, the net surface charge of adsorbent is positive and the adsorption of anions dominates, while, at pH > pH_PZC_, the net surface charge is negative and thus allows the trapping of cations^66^. The pH_PZC_ of the synthesized Fe_3_O_4_ MNPs in the presence of *C. myxa* leaf extract was found to be 7.1 (see Fig. S3, Supporting information). MB is a cationic dye with pK_a_ = 3.8 and has a permanent positive charge in the studied pH range. Hence, at pH lower than pH_PZC_, the adsorption of MB onto Fe_3_O_4_ MNPs decreases due to the positive charge of adsorbent and electrostatic repulsion. In addition, the H^+^ concentration, which is high at lower pH, competes with the positively charged MB for vacant adsorption sites. Thus, at low pH, the adsorption is very low. However, at pH higher than pH_PZC_, the surface charge of MNPs is negative, due to the deprotonation of carboxyl groups and adsorption of OH^−^ on the surface of the adsorbent, and the adsorption of MB increases due to electrostatic attraction between adsorbent and MB. These results confirm that electrostatic attraction plays a key role in the adsorption process. The maximum adsorption is achieved at a pH close to the pH_PZC_ of the adsorbent^63,67^, which explains the optimum pH (= 7.5).

#### Effect of the amount of Fe_3_O_4_ MNPs

Since the adsorption of MB takes place at the Fe_3_O_4_–H_2_O interface, the amount of adsorbent has a great influence on the adsorption capacity. According to Fig. 6b, increasing the amount of adsorbent from 0.17 to 0.50 mg ml^−1^ increases the removal of MB (10 mg L^−1^) from 51 to 88%. This is related to the increased surface area of the adsorbent and access to a large number of adsorption sites for MB^68^. The removal efficiency remains constant at higher amounts of adsorbent (0.67 and 0.83 mg ml^−1^).

#### Effect of the initial concentration of MB

Figure 6c shows the influence of the initial dye concentration on the removal efficiency of MB at a fixed dosage of Fe_3_O_4_ MNPs (0.50 mg ml^−1^) and at pH 7.5. Enhancing the concentration of MB from 5.0 to 12.0 mg L^−1^ did not affect the removal efficiency. However, a further increase of the MB concentration to 15.0 mg L^−1^ clearly reduced the removal efficiency. This can be justified by the saturation of the MB binding sites: at a given dosage of MNPs, the number of MB binding sites is constant. Hence, with the increase in MB concentration, the adsorption of MB molecules becomes a competitive process, which leads to a decrease in removal percentage.

#### Reusability of the synthesized Fe_3_O_4_ MNPs

**Figure 7:**
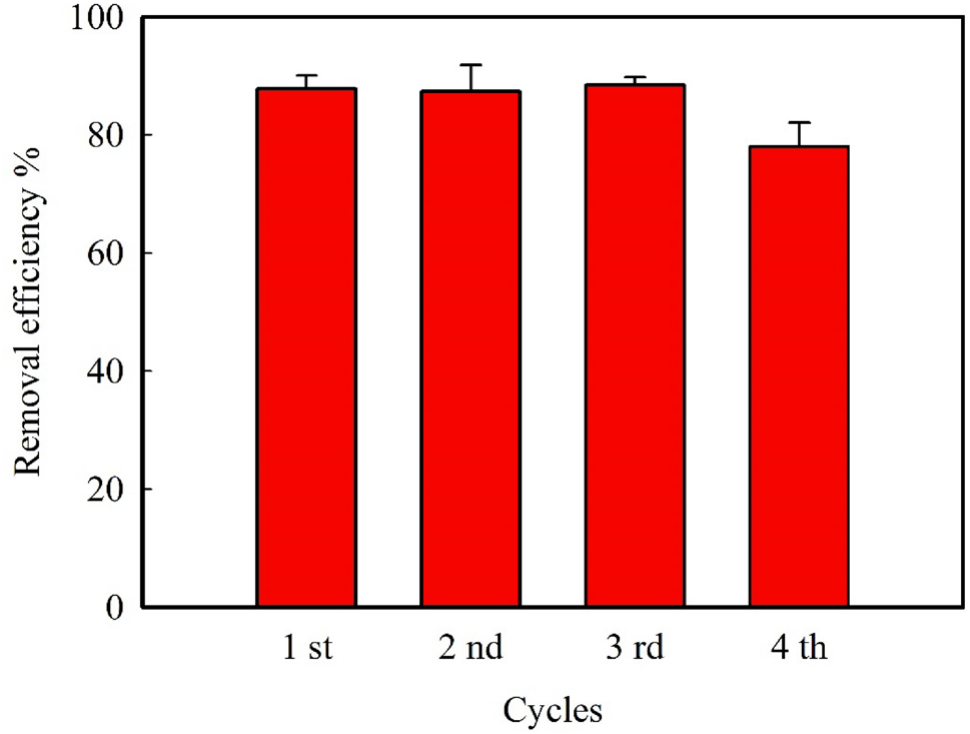
Reusability of the synthesized Fe_3_O_4_ MNPs at the optimum condition (MNPs dosage = 0.50 mg ml^−1^, solution pH = 7.5, and contact time = 60 min).

For any adsorbent, it is desirable that its adsorption capacity remains more or less constant during regenerated and reuse. This property makes the use of the adsorbent economically sustainable, which is especially important for commercial and industrial applications. Therefore, in each cycle, after the adsorption process, the utilized Fe_3_O_4_ MNPs are magnetically separated from the solution, washed with ethanol to remove the adsorbed MB, dried, and reused in the next cycle. From Fig. 7, it can be concluded that the removal efficiency remained more or less constant after three successive runs. After the 4^th^ cycle, only about 11% of its removal efficiency was lost, indicating the stability and reusability of the Fe_3_O_4_ MNPs.

#### Kinetics of dye adsorption

Most adsorption processes are time-dependent. In this study, equilibrium was reached after about 60 min, after which the adsorption efficiency remained almost constant (Fig. 6). Kinetic models are used to investigate different mechanisms controlling the adsorption of the dye molecules from the aqueous solutions; that is, these models help to describe the adsorption rate of solutes from the solute-solution interface. In fact, adsorption kinetics provide valuable information for the design of the adsorption process for practical applications. To investigate the kinetic parameters of the adsorption process in more detail, three kinetic models were considered: pseudo-first-order or Lagergren model (shown in Eq. 4)^69,70^, pseudo-second-order (shown in Eq. 5)^71^, and intraparticle diffusion (shown in Eq. 6)^72,73^.

4 $${\text{log}}\left({q}_{e}-{q}_{t}\right)={\text{log}}({q}_{e})-(\frac{{k}_{1}}{2.303})t$$

5 $$\frac{t}{{q}_{t}}=\frac{1}{{k}_{2}{q}_{e}^{2}}+\frac{t}{{q}_{e}}$$

6 $${q}_{t}={k}_{id}{t}^\frac{1}{2}+C$$

**Figure 8:**
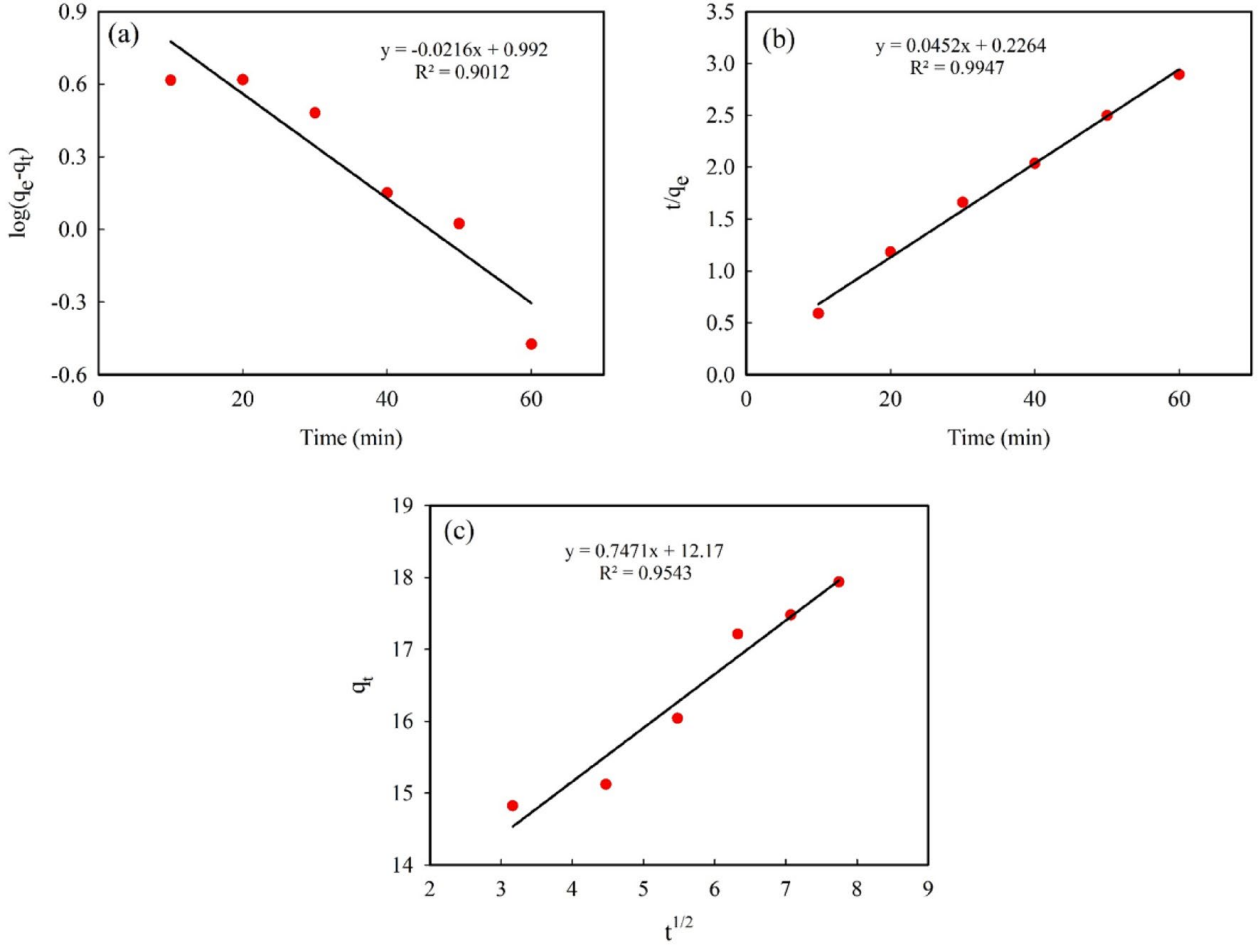
Kinetics of the MB adsorption reaction, (**a**) Pseudo first-order, (**b**) Pseudo second-order, and (**c**) Intraparticle diffusion (experimental conditions: concentration of MB‏ = 12 mg L^−1^, MNPs dosage = 0.50 mg ml^−1^, and pH = 7.5).

The parameter *q*_*e*_ (mg/g) is the adsorption capacity in the equilibrium and *q*_*t*_ is the adsorption capacity at time *t*. *q*_*e*_ and *q*_*t*_ show the amount of MB adsorbed on the magnetic adsorbent. The *k*_*1*_ (min^-1^), *k*_*2*_ (mg/g min), and *k*_*id*_ (mg/g min^0.5^) are rate constants for pseudo-first-order kinetics, pseudo-second-order kinetics, and intraparticle diffusion, respectively^69,71,72^. The *C* (mg/g) in Eq. (6) is a constant value related to the thickness of the boundary layer. Kinetic studies of the adsorption process were performed with an initial concentration of MB‏ of 12 mg L^-1^, Fe_3_O_4_ MNPs dosage of 0.50 mg ml^-1^, and pH 7.5. All the kinetic parameters were calculated by fitting the experimental data to different kinetic models and are presented in Table 1 and Fig. 8. The highest R^2^ value (above 0.99) from fitting the experimental data to the pseudo-second-order kinetic shows that this model describes the process in the best way compared to the others. Moreover, the *q*_*e*_ value calculated from the second-order model is close to the experimental value and demonstrates a smaller deviation compared to the first-order model, which further confirming that the adsorption mechanism is second order. The verification of this model suggests that both adsorbent and adsorbate concentrations are associated with the rate-determining step of the adsorption process along with chemisorption, via valence forces through the exchange or sharing of electrons between the dye and nanoparticles, chelation, coordination and/or complexation^74,75^.

**Table 1:** Kinetics constants for pseudo-first, pseudo-second order and intraparticle diffusion models.

Model				
Pseudo-first order	q_e_ Exp. (mg/g)	q_e_ Cal. (mg/g)	k_1_ (min^−1^)	R^2^_1_
	21.07	9.82	0.050	0.9012
Pseudo-second order	q_e_ Exp. (mg/g)	q_e_ Cal. (mg/g)	k_2_ (mg/g min)	R^2^_2_
	21.07	22.12	0.009	0.9947
Intraparticle diffusion	–	C (mg/g)	k_id_ (mg/g min^0.5^)	R^2^_3_
	–	12.17	0.747	0.9543

#### Adsorption isotherm

The analysis of the adsorption equilibrium models can provide useful information about the adsorption mechanism, surface properties, and affinity of the applied adsorbent^76^. In order to understand the interaction behavior between adsorbate (MB dye) and adsorbent (green synthesized Fe_3_O_4_ MNPs), isotherm experiments were conducted at room temperature and the obtained results were studied with Langmuir and Freundlich models as two common. The Langmuir isotherm is suitable for monolayer adsorption on a surface containing a finite number of identical sites. This model assumes uniform adsorption energies on the surface and there is no transmigration of the adsorbate in the plane of the surface. During the adsorption process, an active site adsorbs a dye molecule and then does not allow any additional adsorption on the occupied active site^76–78^. The linearized Langmuir isotherm is expressed as^74^:

7 $$\frac{{C}_{e}}{{q}_{e}}=\frac{1}{b{q}_{max}}+\frac{{C}_{e}}{{q}_{max}}$$

where *q*_*e*_ is the equilibrium adsorption capacity as denoted previously, *C*_*e*_ (mg L^−1^) is the equilibrium concentration of MB in solution, *q*_*max*_ (mg g^−1^) is the maximum adsorption capacity, and *b* (L mg^−1^) is the Langmuir constant.

The Freundlich model assumes that with an increase in the concentration of the adsorbate, the adsorbate concentration on the adsorbent surface also increases and, correspondingly, the sorption energy decreases exponentially with the completion of adsorption sites of adsorbent. On the other hand, this isotherm was used to describe the adsorption characteristics of multilayer and heterogeneous surfaces with unequally available adsorption sites that have different adsorption energies^76,77^. The Freundlich adsorption isotherm is given as^74^:

8 $$ln{q}_{e}=ln{k}_{f}+\frac{1}{n}ln{C}_{e}$$

where *n* and *k*_*f*_ are the Freundlich adsorption isotherm constants related to the adsorption intensity and the adsorption capacity, respectively.

**Figure 9:**
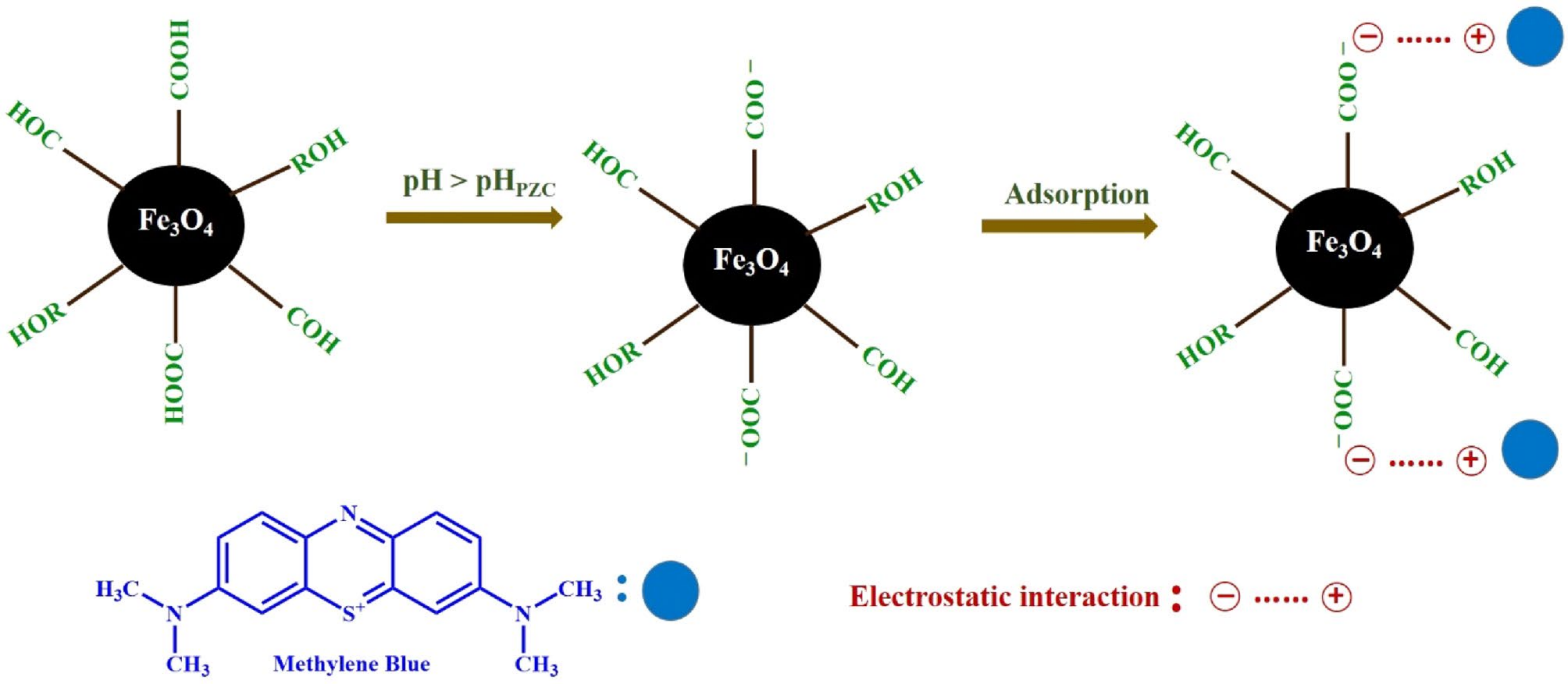
Illustration of the proposed mechanism for adsorption of MB by synthesized Fe_3_O_4_ MNPs.

The obtained Freundlich and Langmuir constants from regression analysis are represented in Table 2. Based on the R^2^ values, the Langmuir equation describes the adsorption of MB onto Fe_3_O_4_ MNPs better than the Freundlich equation. Herein, the *q*_*max*_ of the MNPs was found to be 17.79 (± 0.06) mg g^−1^ in three repeated sets of experiences. Based on the obtained results, it is clear that the modification of MNPs with the phytochemical compounds enhanced the adsorption capability of adsorbent. To show the importance of modifying the surface of MNPs with the *C. myxa* leaf extract in the adsorption capacity, the *q*_*max*_ of bare MNPs was calculated in similar conditions and it was found that in spite of weak correlation Langmuir model, the *q*_*max*_ was significantly lower than *q*_*max*_ of modified MNPs (< 65%). The important role of functional groups obtained from the extract is schematically shown in Fig. 9.

**Table 2:** Adsorption isotherm constants for binding of MB to the synthesized Fe_3_O_4_ MNPs.

Langmuir			Freundich		
q_max_	b	R^2^	k_f_	n	R^2^
17.79	0.44	0.9979	22.69	6.88	0.8749

#### Comparison with other adsorption studies using Fe_3_O_4_ MNPs

The commercial applicability of an adsorbent depends on its adsorption capacity, specific surface area, availability, and compatibility with the environment and the user. Table 3 shows a comparison of the adsorption capacity of various green synthesized Fe_3_O_4_ MNPs reported in the literature^79–82^ for the removal of MB from aqueous solutions. Based on Table 3, Fe_3_O_4_ MNPs prepared with *C. myxa* leaf extracts have better or comparable adsorption capacity compared with other MNPs, with the exception of MNPs prepared with Cress seed mucilage. Therefore, the synthesized Fe_3_O_4_ MNPs produced with *C. myxa* leaf extract appear to be a sustainable adsorbent for the removal of MB from aqueous solutions. It is clear that *q*_*max*_ is one of the most important criteria for the application of nanosorbents in real applications. On the other hand, by comparing the required pH of our suggested MNP with previous green synthesized ones, it can be observed from Table 3 that the pH of the current work is not too alkaline or acidic which makes it a good applied sorbent for application without serious need of pH adjustment. The highlight point of the current work was the using *C. myxa* leaf to enhance the surface properties of Fe_3_O_4_ MNPs which changes its adsorption ability in comparison with the previous similar reports.

**Table 3:** Comparison of various plant synthesized Fe_3_O_4_ MNPs as adsorbent for the removal of MB from aqueous solution with proposed Fe_3_O_4_ MNPs adsorbent.

No	Plant soueces	q_max_ (mg g^−1^)	pH	References
1	*Zanthoxylum armatum* leaf	10.47	11.0	^79^
2	*Ficus hispida L.* leaf	16.39	11.0	^80^
3	Cress seed mucilage	44.61	4.0	^81^
4	Green tea	7.25	7.0	^82^
5	*Cordia myxa* leaf	17.79 (± 0.06)	7.5	This work

## Conclusions

Here, we reported a green, one-pot synthesis of Fe_3_O_4_ MNPs using *C. myxa* leaf extract. The resulting spherical particles had a size of 21–32 nm with a saturation magnetization value of about ~ 49.48 emu/g. XRD analysis of Fe_3_O_4_ MNPs confirmed their cubic spinel structure. FT-IR spectroscopy confirmed the presence of *C. myxa* phyto-compounds in the nanoparticles, contributing to their stabilization. BET revealed the mesoporous nature of the synthesized Fe_3_O_4_ MNPs with a high surface area (~ 115.07 m^2^/g) that could act as an effective adsorbent to remove MB from aqueous media. The maximum removal efficiency (88.8%) was recorded at the following optimum operation conditions; adsorbent dosage = 0.50 mg ml^−1^, solution pH = 7.5, and contact time = 60 min. with a maximum adsorption capacity of 17.79 mg/g at pH 7.5, after 60 min with pseudo-second-order kinetics. The maximum adsorption capacity of 17.79 mg/g was recorded from the Langmuir model. The second-order isotherm model shows the best fit with the experimental data. It was demonstrated that modification and capping of MNPs with the phytochemical compounds present in *C. myxa* leaf extract increases the adsorption capability of the adsorbent. The Fe_3_O_4_ MNPs were easily recovered from the solution by an external magnet and could be successfully be reused several times and no significant decrease in removal performance was observed. The non-toxic and magnetically separable green synthesized Fe3O4 MNPs could be applied as a cost-effective adsorbent with possible wide application in wastewater treatment technologies and removal of organic water pollutants. However, the study of the thermodynamics of the adsorption process was not within the scope of this work but its investigation in future studies can enhance our knowledge about the adsorption property of the proposed nanosorbent. The presented work was not the best in comparison with the previous studies but can obtain an alternative way to decrease the dye pollution in aqueous media using stable and green-stabilized MNPs which are simply and easily prepared.

### Supplementary Information

Supplementary Figures.

## Data Availability

Most of details and data have been included in the manuscript and other details can be available via a reasonable request to the corresponding author (F. Samari).
