# Supplementary material for: A facile strategy for preparation of Fe3O4 magnetic nanoparticles using Cordia myxa leaf extract and investigating its adsorption activity in dye removal
Source: Sci Rep. 2024 Jan 2;14:84. doi: 10.1038/s41598-023-50550-1 (PMC10762222; doi:10.1038/s41598-023-50550-1)
Supplement: Supplementary file 1 — Supplementary Figures. [file 41598_2023_50550_MOESM1_ESM.docx]

**A facile strategy for preparation of Fe_3_O_4_ magnetic nanoparticles using *Cordia myxa* leaf extract and investigating its adsorption activity in dye removal**

Elham Ghoohestani*^a^*, Fayezeh Samari*^a^*^,^*^b^*^*^, Ahmad Homaei*^c^*, Saeed Yosuefinejad*^d^*

*^a^ Department of Chemistry, Faculty of Sciences, University of Hormozgan, Bandar Abbas, Iran*

*^b^ Nanoscience, Nanotechnology and Advanced Materials Research Center, University of Hormozgan, Bandar Abbas, Iran*

*^c^ Department of Marine Biology, Faculty of Marine Science and Technology, University of Hormozgan, Bandar Abbas, Iran*

*^d^ Research Center for Health Sciences, Institute of Health, Department of Occupational Health Engineering, School of Health, Shiraz University of Medical Sciences, Shiraz, Iran,*

*Corresponding authors: Fayezeh Samari, Department of Chemistry, Faculty of Sciences, University of Hormozgan, Bandar Abbas, Iran. P.O. Box 3995, Tel.: +98 76 33711000-11; Fax: +98 76 33670716, E-mail address: [fsamari@hormozgan.ac.ir](mailto:fsamari@hormozgan.ac.ir) (F. Samari)

**Short Title:**

**Fe_3_O_4_ magnetic nanoparticles using *Cordia myxa* leaf extract**

**
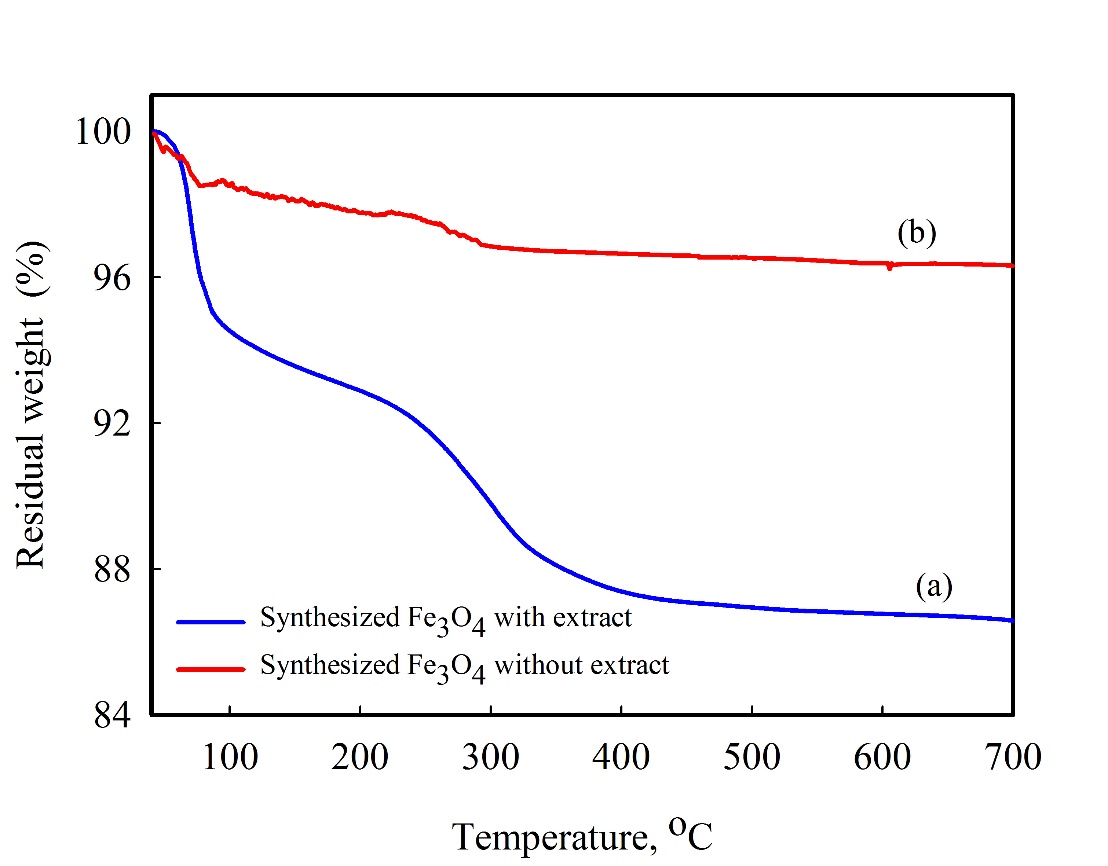
**

**Figure S1.** Thermogravimetric analysis curve of synthesized Fe_3_O_4_ MNPs (a) with and (b) without the extract.

**
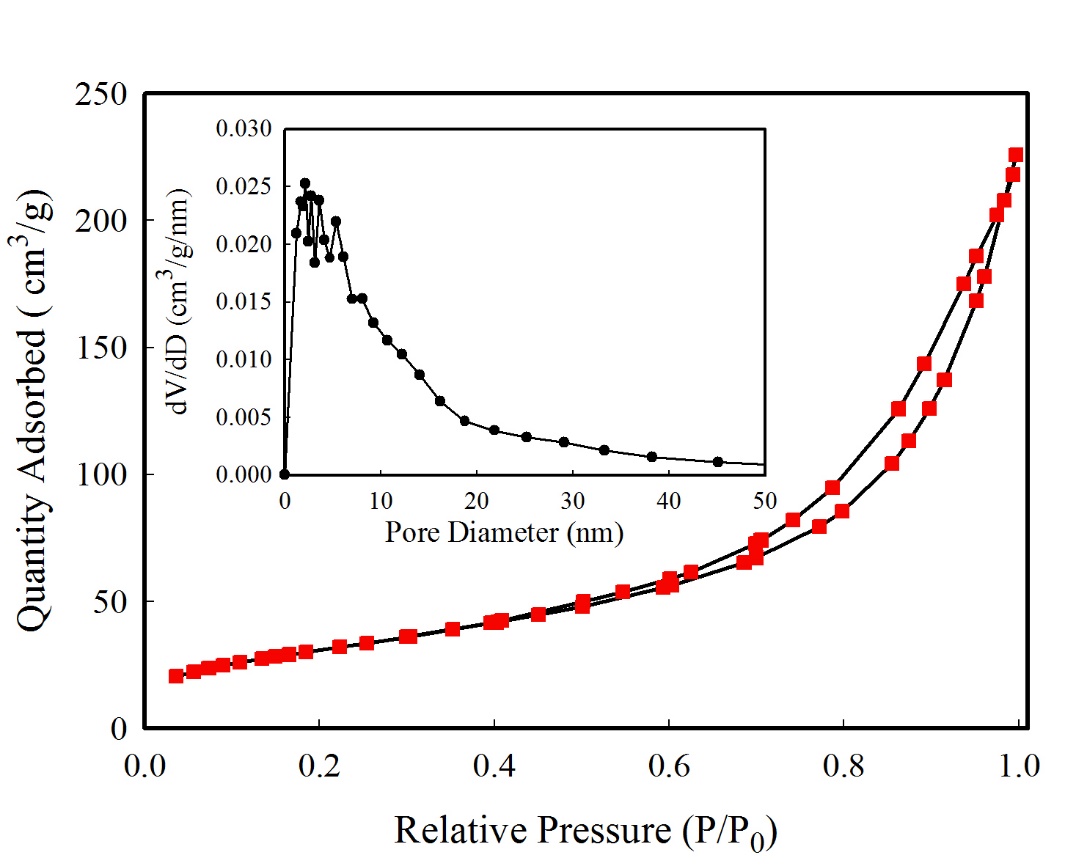
**

**Figure S2.** Nitrogen adsorption and desorption isotherms, and pore size distribution (inset) of the synthesized Fe_3_O_4_ MNPs.

**
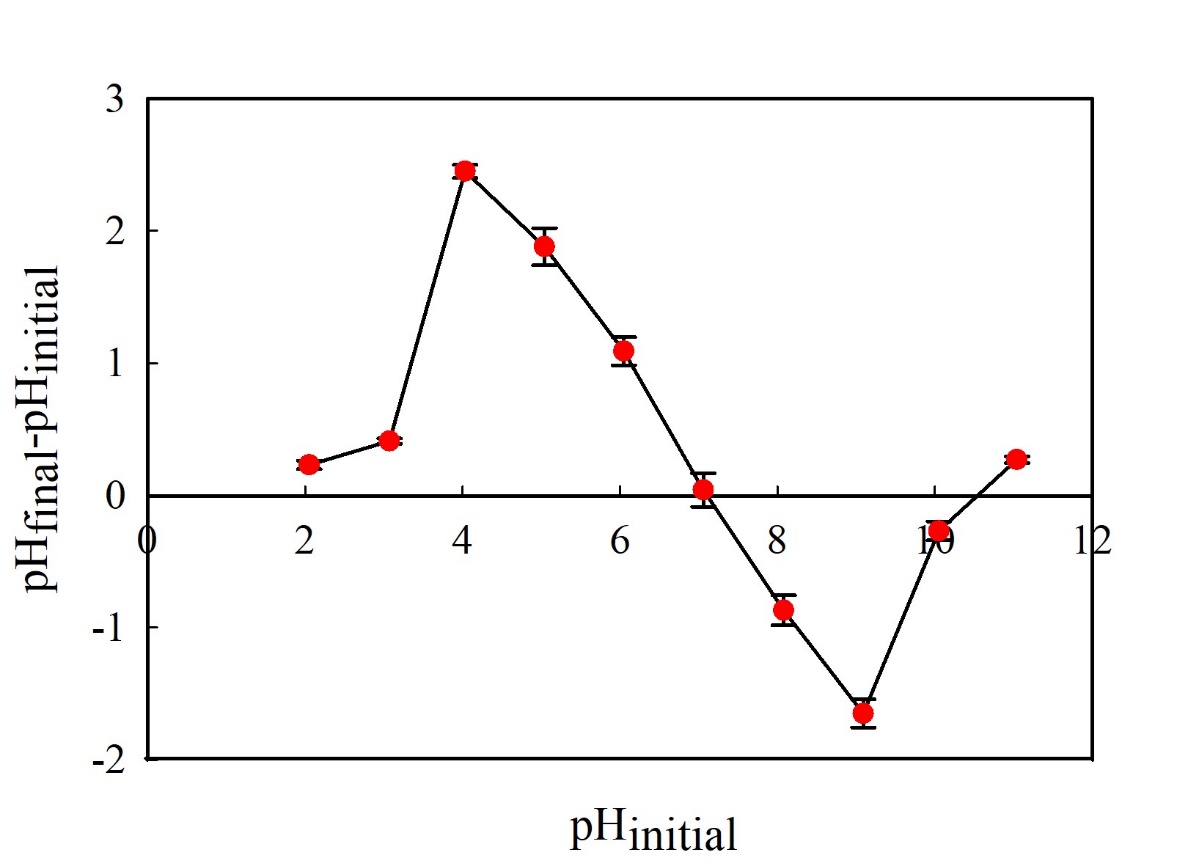
**

**Figure S3.** Determination of the pH_PZC_ of the synthesized Fe_3_O_4_ MNPs by the pH drift method.
